# Case report: Reversible splenial lesion syndrome preceding the onset of multiple sclerosis

**DOI:** 10.3389/fimmu.2024.1517719

**Published:** 2025-01-07

**Authors:** Matthias Mauritz, Dariia Kliushnikova, Ferdinand Otto, Andrea Harrer, Tobias Moser, Richard Friedrich Radlberger, Waltraud Kleindienst, Eugen Trinka, Peter Wipfler

**Affiliations:** ^1^ Department of Neurology, Christian Doppler University Hospital, Paracelsus Medical University and Centre for Cognitive Neuroscience, Salzburg, Austria; ^2^ Department of Dermatology and Allergology, Paracelsus Medical University, Salzburg, Austria; ^3^ Department of Public Health, Health Services Research and Health Technology Assessment, UMIT – University for Health Sciences, Medical Informatics and Technology, Hall in Tirol, Austria; ^4^ Neuroscience Institute, Christian Doppler University Hospital, Paracelsus Medical University, Salzburg, Austria

**Keywords:** reversible splenial lesion syndrome, neuroimmunology, multiple sclerosis, neuroinfectiology, multiple sclerosis pathogenesis

## Abstract

**Background:**

The reversible splenial lesion syndrome is frequently associated with systemic and central nervous system infections. Whether an infection associated with the occurrence of the reversible splenial lesion syndrome could play a role in the later development of multiple sclerosis is unknown.

**Methods:**

Case Report.

**Results:**

A 27-year-old woman developed an infection-related reversible splenial lesion syndrome. Diagnostic findings did not establish a specific type of infection, but revealed evidence for a potential disposition towards autoimmunity. 32 months after the initial presentation, new clinical and radiological manifestations developed that led to a diagnosis of multiple sclerosis.

**Conclusions:**

In susceptible individuals, infectious disease processes involving the central nervous system, such as described in this case, might be a factor in the pathogenesis of multiple sclerosis. More research on the prodromal stage of multiple sclerosis is needed to better understand the relationship between infections and autoimmunity.

## Introduction

The term reversible splenial lesion syndrome (RESLES) refers to a rare but distinct neuroradiological entity characterized by the magnetic resonance imaging (MRI) presentation of a single lesion in the splenium of the corpus callosum (SCC) that completely resolves on follow-up imaging within a few weeks (1).

RESLES lesions are typically located in the center of the splenium, have an ovoid or round-shaped appearance and present with increased signal on isotropic diffusion-weighted imaging (DWI) together with abnormally low signal on apparent diffusion coefficient (ADC) maps, high signal intensity on T2-weighted imaging and no signs of gadolinium contrast enhancement. The occurrence of RESLES in heterogeneous clinical conditions, most frequently in association with antiseizure medication withdrawal, seizures, systemic and central nervous system (CNS) infections and metabolic disturbances such as hypoglycemia, amongst others, has precluded firm conclusions about its underlying pathogenesis (1). While the abnormal increase in proton-diffusivity restriction suggests cytotoxic (intracellular) edema, the reversibility of the lesion as well as findings from studies using advanced neuroimaging argue for RESLES lesions to represent an intercellular intramyelinic edema (2–4). In the view of existing hypotheses, the edema constitutes a common pathophysiological endpoint to processes such as fluid-balance alterations or inflammatory cascades (5).

RESLES has been reported in association with a wide range of systemic and CNS infections of both viral and bacterial origin. In many of these cases, patients suffered from transient neurological symptoms but made a full clinical recovery, prompting the name mild encephalitis/encephalopathy with reversible splenial syndrome (MERS) (6).

Originally reported only in children from East Asia, case reports later described MERS in adult patients worldwide (7). In nearly all cases fever was part of the initial presentation. The most frequent neurological manifestation were disorders of consciousness and many patients had a mild cerebrospinal fluid (CSF) pleocytosis and hyponatremia. Pathogens reported as the cause of infections associated with MERS cases have included, amongst others, influenza virus, rota virus, adenovirus, parainfluenza virus, measles virus, parvovirus B19, rubella virus, cytomegalovirus, mycoplasma pneumoniae, legionella pneumophila, streptococcus pneumoniae and salmonella enteritidis (5).

Infections can cause acute neurological disease via direct infiltration of the nervous system or indirectly via effects of systemic inflammation, cross reaction and metabolic derangements. In addition, infectious pathogens are thought to play an important role as environmental triggers in the complex pathogenesis of autoimmunity.

One of the strongest associations between anteceding infections and the subsequent development of neurological autoimmune disease has been demonstrated for the Guillain-Barré Syndrome (GBS). Other examples of neurological disease recognized as a potential postinfectious syndrome include autoimmune encephalitis or acute disseminated demyelinating encephalomyelitis (ADEM) (8).

Contrary to acute postinfectious neurological diseases such as GBS, the role of infections is less clear in the pathogenesis of chronic autoimmune-mediated neurological diseases. One such disease is multiple sclerosis (MS), which is the most prevalent chronic inflammatory CNS disorder (9). Its autoimmune-mediated disease process has led to infections being discussed as possible disease triggers in genetically predisposed individuals. Whether an acute infection that causes neurological symptoms as in MERS might have a role in the future development of an autoimmune-mediated CNS disease such as MS is currently unclear.

## Case report

**Figure 1:**
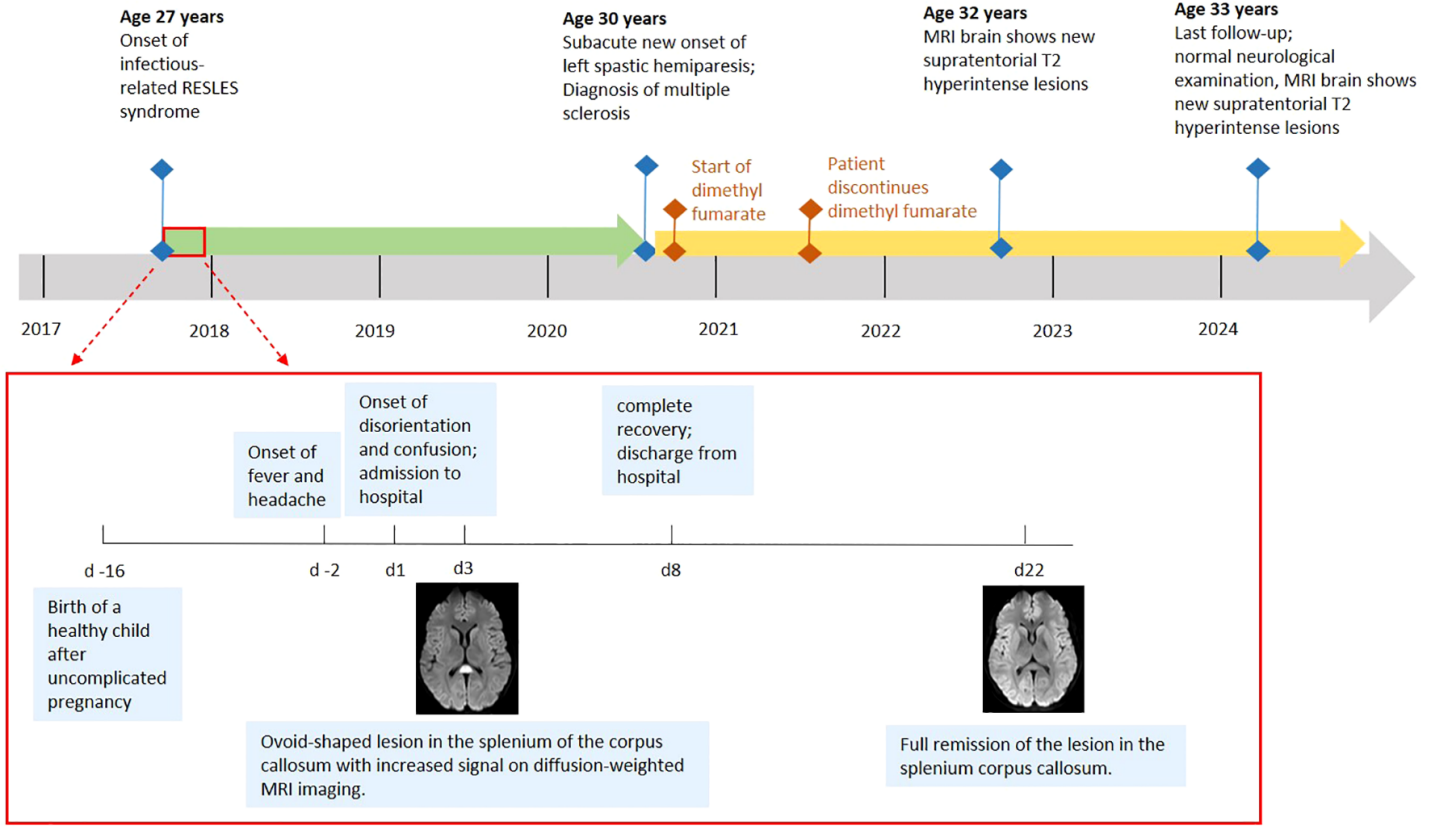
Timeline of clinical events.

**Figure 2:**
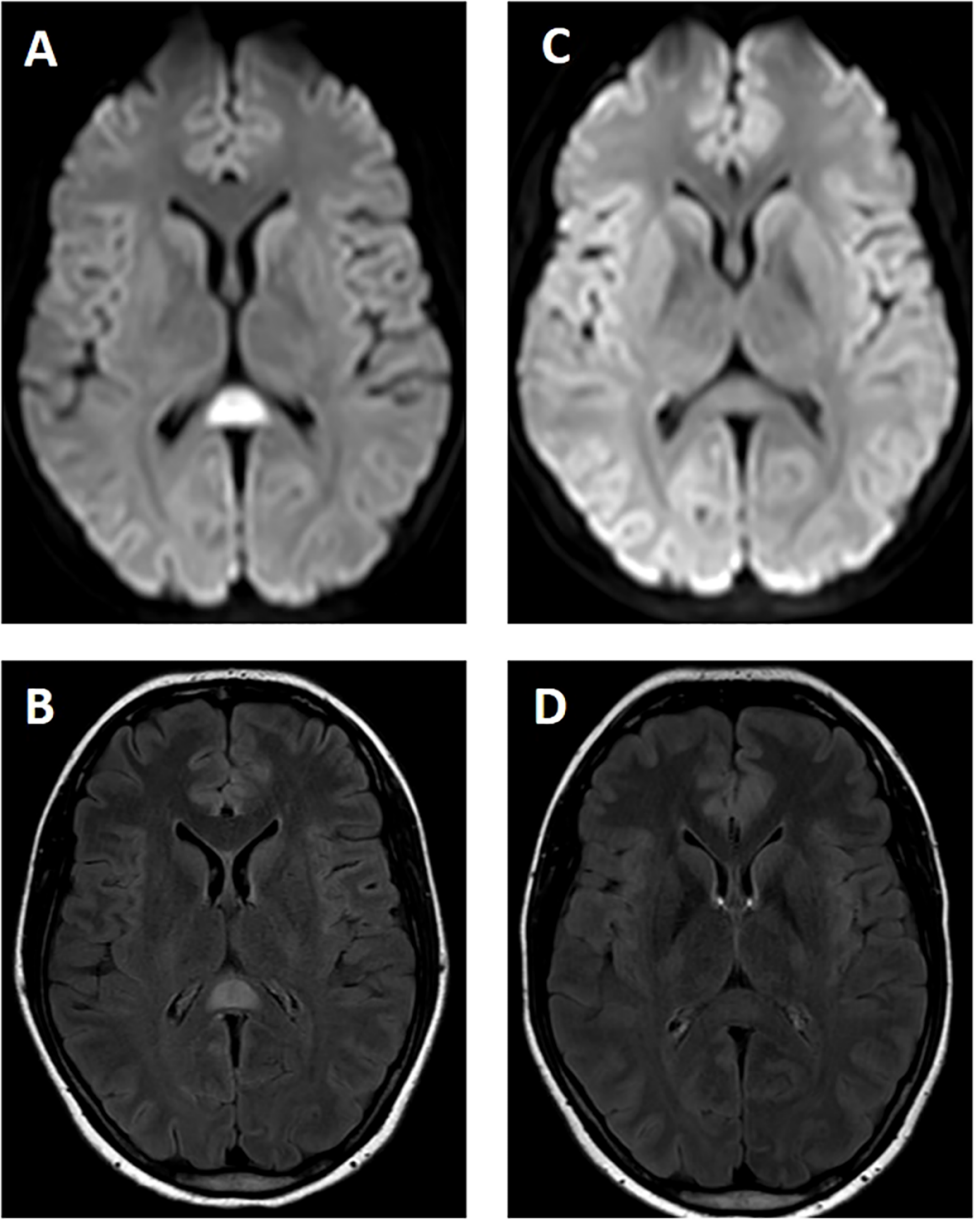
**(A, B)** MRI of the brain on day 3. A single, ovoid-shaped lesion is seen in the splenium of the corpus callosum with increased signal on diffusion-weighted imaging **(A)** and fluid inversion recovery sequences **(B)**. **(C, D)** MRI of the brain on day 22 shows full remission of the lesion in the splenium corpus callosum.

A 27-year-old woman with an unremarkable previous medical history presented with altered mental status after two days of fever and headache (**Figure 1**). Sixteen days before, the patient had given birth to a healthy child after an uneventful pregnancy. There was no family history of MS or any other neurological disease. On clinical examination, disorientation, reduced attention, psychotic thoughts as well as a temporal temperature of 39°Celsius were noted. MRI of the brain, performed on day one and day three, revealed a single, ovoid-shaped lesion in the center of the SCC with a hyperintense signal on both T2-weighted imaging and DWI as well as low ADC map values (**Figures 2A, B**). No contrast enhancement was seen within the SCC lesion. There were no other lesions visible elsewhere in the brain. Electroencephalography (EEG) showed normal background activity and did not reveal any epileptiform activity. Elevated blood levels for C-reactive protein (CRP) and lactate dehydrogenase were notable as was moderate hypokalemia and hypochloremia (**Supplementary Table 1**). The blood levels of all other electrolytes, glucose, thyroid-stimulating hormone as well as coagulation-, liver- and kidney function tests were normal. CSF analysis revealed mild lymphocytic pleocytosis (**Table 1**). While the CSF/serum albumin ratio was within normal range, there was evidence for intrathecal immunoglobulin synthesis. The diagnostic work-up could not identify an infectious agent (**Supplementary Table 2**). Splenomegaly was noted on abdominal ultrasound. Serum IgG antibodies against Epstein-Barr-Virus (EBV) were present, but IgM EBV antibodies were negative. Testing for antineuronal and paraneoplastic autoantibodies was negative (**Supplementary Table 2**). Empirical treatment with aciclovir and ceftriaxone was administered. The patient made a full clinical recovery and was discharged by day eight. Brain MRI on day 22 showed complete resolution of the SCC lesion and did not detect any new lesions (**Figures 2C, D**).

**Table 1:** Cerebrospinal fluid analysis results, RESLES presentation and MS presentation.

Cerebrospinal fluid (CSF) Variable	Value, RESLES Presentation	Value, MS Presentation	Reference Range
White cell count (per μl)	6	8	0-4
Erythrocytes (per μl)	0	0	0
Protein (mg/dl)	21	25	3-50
Glucose (mg/dl)	60	63	40-80
Lactate (mmol/l)	2.0	1.4	1.1-2.4
Cytology	Lymphocytes and Monocytes	Lymphocytes and Monocytes	
CSF albumin (mg/dl)	10.6	15.7	13.9-50
CSF/Serum albumin quotient	3.2	3.6	< 6.5
CSF IgG (mg/dl)	2.76	3.76	0.48-5.86
CSF/Serum IgG quotient	2.7	3.0	
Intrathecal IgG Synthesis			
Intrathecal fraction (Reiber)	22.0%	20.0%	0.0
IgG Index	0.84	0.83	< 0.7
Oligoclonal bands	Not tested	positive	negative

**Figure 3:**
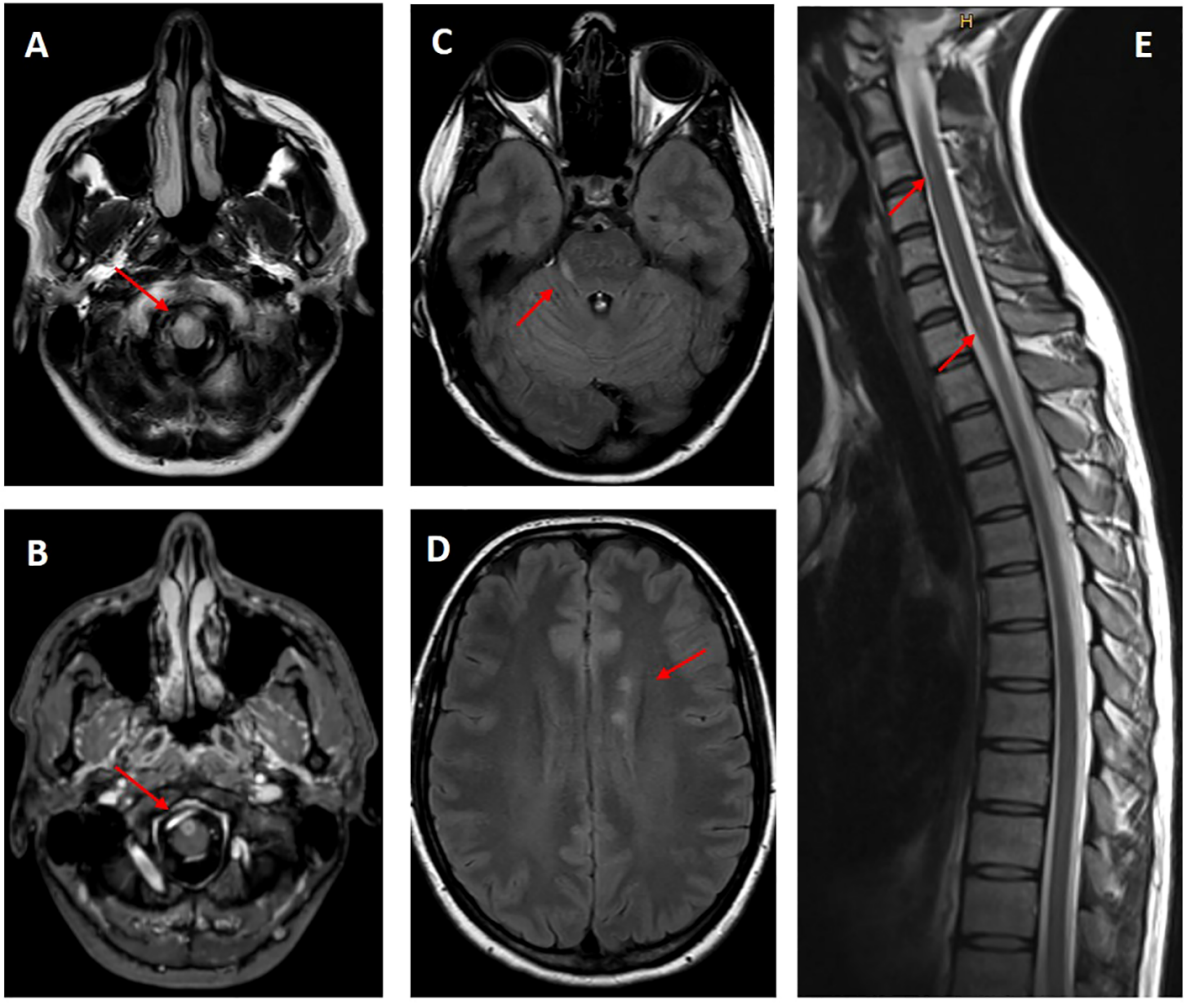
MRI of brain and cervical spine, MS presentation. New T2-hyperintense lesions are seen, amongst others, in the right paramedian medulla oblongata **(A)**, in the right inferior cerebellar peduncle **(C)**, in the periventricular white matter **(D)** and in the cervical spinal cord **(E)**. Contrast enhancement is seen, amongst others, with the lesion in the right paramedian medulla oblongata **(B)**.

Thirty-two months later, the patient returned with a mild, left-sided, spastic hemiparesis of subacute onset. Brain MRI revealed a T2-hyperintense, round-shaped, contrast enhancing lesion in the right paramedian medulla oblongata (**Figures 3A, B**). Multiple other new T2-hyperintense lesions located in the body of the corpus callosum, in the right superior cerebellar peduncle (**Figure 3C**), in the periventricular white matter (**Figure 3D**) and juxtacortical to the left gyrus rectus, were detected. Of these, one periventricular lesion and the lesion juxtacortical to the left gyrus rectus showed gadolinium enhancement. In addition, two small, T2-hyperintense, non-enhancing lesions were identified in the cervical spinal cord (**Figure 3E**). A mild lymphocytic pleocytosis and positive oligoclonal bands were found on CSF analysis (**Table 1**). A diagnosis of MS was made and high-dose parenteral methylprednisolone was administered for three days. Full remission of the neurological deficits occurred within a few weeks. Treatment with dimethyl fumarate was started, but was discontinued by the patient after eleven months. At the last follow-up 76 months after the initial presentation, the patient reported no new symptoms and the neurological examination did not reveal any abnormalities. However, MRI of the brain did show multiple new supra- and infratentorial white matter lesions.

## Discussion

The development of MS years after an episode of RESLES is an intriguing clinical observation. First, it is necessary to state that the occurrence of a RESLES episode and the development of MS thereafter could only be mere coincidence. However, it is tempting to discuss questions about potential associations that are raised by this case.

Could an episode of RESLES be the first manifestation of MS? Transient lesions of the splenium have previously been described in association with a variety of disorders, but there have been no reports linking them to MS (1). While in general lesions in the corpus callosum (CC) are commonly found in MS, the appearances of MS-related CC lesions are distinctive from the typical RESLES lesion. They typically either present as T2-hyperintense band-like lesions at the undersurface of the CC from which in a perpendicular orientation thin, linear T2-hyperintense lesions radiate into the CC (subcallosal or subependymal striations) or as T2-hyperintense, ovoid lesions that radiate in a perivenular distribution from the ventricular surface of the CC perpendicularly into the pericallosal white matter (Dawson fingers) (10). MS-related CC lesions can show diffusion restriction, but in contrast to RESLES lesions they can also show contrast enhancement and are usually unlikely to disappear over time. The SCC lesion in this case did display all the characteristics of a typical RESLES lesion, which as described above most likely corresponds to an intramyelinic edema. Particularly the rapid resolution of the RESLES lesion would not be in line with true demyelination. Confusion and delirium, as seen in our patient, are the symptoms most frequently encountered with midline splenial lesions, but would represent rather atypical symptoms for an initial MS manifestation (11). The presence of fever is another aspect of the clinical presentation arguing against this RESLES episode being MS-mediated.

Indeed, the clinical findings of fever and raised serum inflammatory markers indicated that the patient´s RESLES episode was associated with a possible acute infection, thus qualifying for the term MERS. The clinical findings did not allow to distinguish with certainty between a systemic infection leading to an encephalopathy and a true CNS infection. While the clinical presentation was in principle compatible with encephalitis, the marked elevation of serum inflammatory parameters together with evidence of splenomegaly and only a very mild CSF pleocytosis suggested a systemic infectious process. A definite infectious focus could not be localized and diagnostic studies from both serum and CSF could not detect a definitive pathogen.

Could an infection, which resulted in the reversible clinical and radiological CNS abnormalities, have played a triggering role for the later development of MS? IgG anti-EBV-antibodies as a prerequisite for MS pathophysiology were already present in our patient at the time of RESLES. The absence of any chronic, typical MS lesions at the time of the acute splenial lesion indicates that an inflammatory demyelinating pathology had not yet been established. Infections have been suspected to induce autoimmunity in genetically susceptible individuals and have also been studied as potential disease triggers in MS. A strong link between MS and EBV infection has by now been established on a population level (12). Infections with other viruses (e.g. human herpesvirus 6) have also been discussed as risk factors for MS (13). Proposed mechanisms of pathogen-induced autoimmunity in MS include antigen-specific responses such as molecular mimicry and epitope spreading that may lead to the generation of autoreactive lymphocytes and immune tolerance breakdown (8). Autoreactive lymphocytes at peripheral sites might then later become activated by an unspecific immune-response against another infection and subsequently migrate to the CNS (14). One might speculate whether in this case the acute infection that occurred in association with RESLES induced such a “bystander activation” in an individual already primed for autoimmunity. However, in our case pathophysiological considerations are limited by the fact that we could not define both the primary focus of infection and a definitive infectious agent during the RESLES episode.

Is the disease, which the patient developed almost three years after the RESLES episode, something different from MS? Our patient had a typical clinical presentation and demographic characteristics, showed characteristic demyelinating lesions with dissemination in time and space and had typical CSF findings. Thus, the 2017 McDonald criteria were fulfilled (15). In addition, there was no evidence for any other disease that would have better explained the clinical presentation than MS.

In summary, we have made the clinical observation of a young adult developing MS three years after an infectious-related RESLES episode. However, much is still unclear about the prodromal stage of MS and considerable research is needed to identify individuals with prodromal symptoms who are at high risk of being diagnosed with MS.

## Data Availability

The datasets presented in this article are not readily available because of ethical and privacy restrictions. Requests to access the datasets should be directed to the corresponding author.

## Supplementary material

Supplementary Table 1 Abnormal laboratory parameters, RESLES presentation.

Supplementary Table 2 Overview of the diagnostic work-up, RESLES presentation.
