# Supplementary figures and images for: Case report: Reversible splenial lesion syndrome preceding the onset of multiple sclerosis
Source: Front Immunol. 2025 Jan 7;15:1517719. doi: 10.3389/fimmu.2024.1517719 (PMC11746013; doi:10.3389/fimmu.2024.1517719)

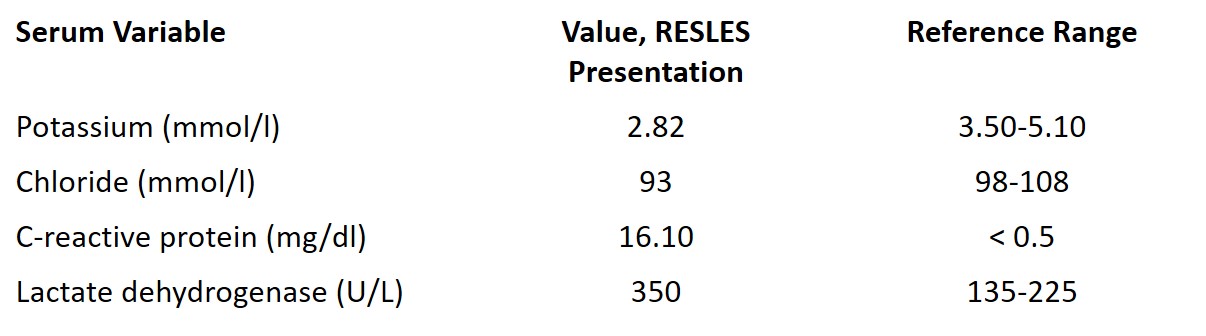

Supplement: Supplementary Table 1 — Abnormal laboratory parameters, RESLES presentation. [file Image1.jpeg]

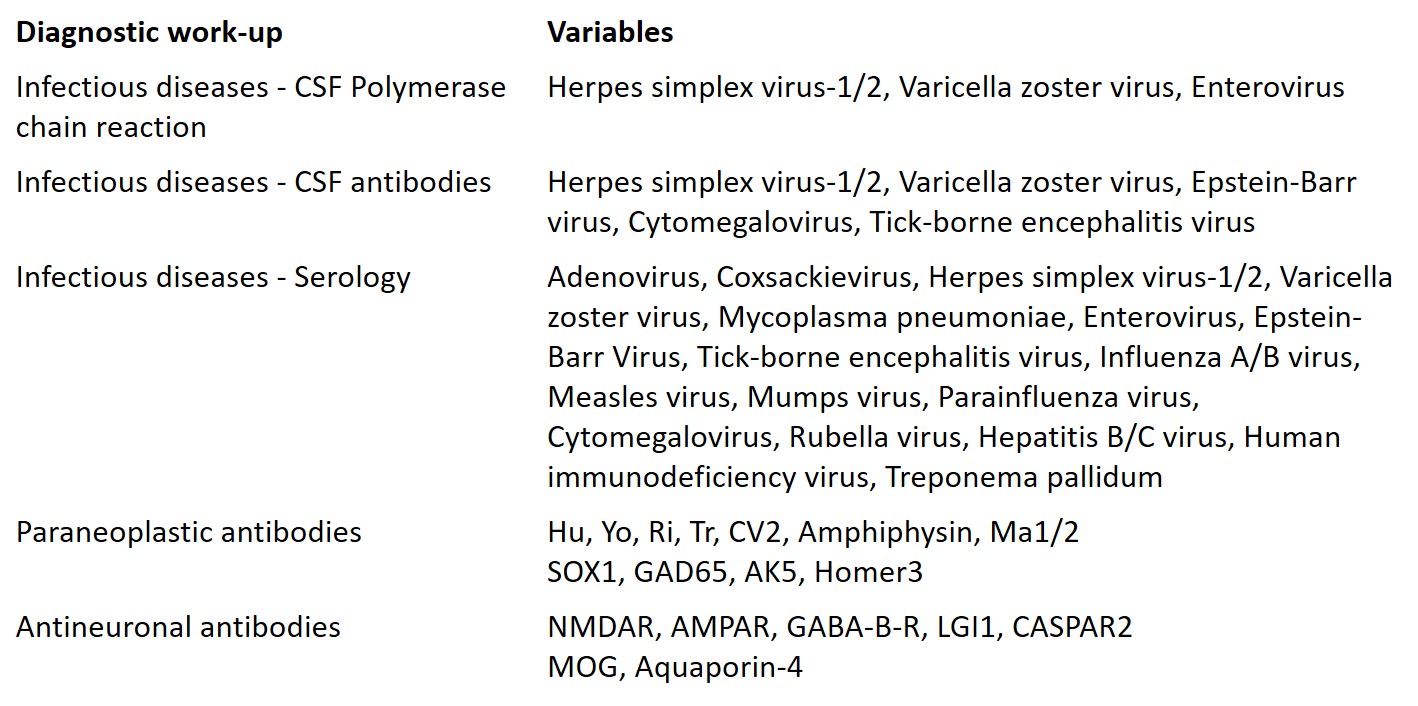

Supplement: Supplementary Table 2 — Overview of the diagnostic work-up, RESLES presentation. [file Image2.jpeg]
